# Following the Water: A Controlled Study of Drinking Water Storage in Northern Coastal Ecuador

**DOI:** 10.1289/ehp.11296

**Published:** 2008-07-07

**Authors:** Karen Levy, Kara L. Nelson, Alan Hubbard, Joseph N.S. Eisenberg

**Affiliations:** 1 Department of Environmental Science, Policy, and Management, University of California, Berkeley, California, USA; 2 Department of Civil and Environmental Engineering, University of California, Berkeley, California, USA; 3 School of Public Health, Division of Biostatistics, University of California, Berkeley, California, USA; 4 School of Public Health, Department of Epidemiology, University of Michigan, Ann Arbor, Michigan, USA

**Keywords:** diarrhea, drinking water quality, Ecuador, microbial contamination, point of use, recontamination, waterborne disease

## Abstract

**Background:**

To design the most appropriate interventions to improve water quality and supply, information is needed to assess water contamination in a variety of community settings, including those that rely primarily on unimproved surface sources of drinking water.

**Objectives:**

We explored the role of initial source water conditions as well as household factors in determining household water quality, and how levels of contamination of drinking water change over time, in a rural setting in northern coastal Ecuador.

**Methods:**

We sampled source waters concurrently with water collection by household members and followed this water over time, comparing *Escherichia coli* and enterococci concentrations in water stored in households with water stored under controlled conditions.

**Results:**

We observed significant natural attenuation of indicator organisms in control containers and significant, although less pronounced, reductions of indicators between the source of drinking water and its point of use through the third day of sampling. These reductions were followed by recontamination in approximately half of the households.

**Conclusions:**

Water quality improved after water was transferred from the source to household storage containers, but then declined because of recontamination in the home. Our experimental design allowed us to observe these dynamics by controlling for initial source water quality and following changes in water quality over time. These data, because of our controlled experimental design, may explain why recontamination has been reported in the literature as less prominent in areas or households with highly contaminated source waters. Our results also suggest that efforts to improve source water quality and sanitation remain important.

Worldwide, 1.1 billion people still did not have access to safe drinking water in 2002 (United Nations 2005), and every day > 6,500 children die from diarrheal illness (Kosek et al. 2003). If we are to move toward the Millennium Development Goals of halving the number of people without access to safe water by 2015 (United Nations 2005), a variety of different interventions may be necessary, because water quality and water use patterns depend on environmental, social, economic, and cultural characteristics of a given area. To design the most appropriate interventions to improve water quality and supply, information is needed to assess the characteristics of water contamination in a variety of contexts and community settings, including those areas that rely primarily on unimproved surface sources of drinking water. In this study, we explored the role of initial source water conditions as well as household factors in determining water quality at the point of use (POU).

Many researchers have observed that storing water in the household leads to a deterioration of water quality because of recontamination in the home. Even if families have a source of clean drinking water, water may become contaminated in the home because of poor hygiene and water-handling practices. Factors known to affect recontamination of water in the home include size of the storage vessel mouth (e.g., Mintz et al. 1995), transfer of water between containers from collection to storage (e.g., Lindskog and Lindskog 1988), hand-to-water contact and dipping of utensils (e.g., Hammad and Dirar 1982; Trevett et al. 2005), and bacterial regrowth within the storage container (e.g., Momba and Kaleni 2002). Studies have also shown that organisms can prosper in biofilms in containers (Jagals et al. 2003).

Wright et al. (2004) carried out a systematic meta-analysis of 57 studies measuring bacteria counts for source water and stored water in the home to assess how contamination varied between different regions and community settings. They concluded that, in general, bacteriologic quality of drinking water significantly declines after collection, although they noted considerable variability between community settings in the extent of this post-collection contamination. For example, they noted less pronounced recontamination in homes with poorer-quality source water. However, few of the studies on stored water followed water in a household over time, and even fewer used proper controls to assess how water quality changes when water was stored outside of the environs of the household. In general, mean source water quality has been simply compared with mean household stored water quality, or household samples have been matched to specific sources, but sample collection has not been matched in time. Studies that have used controls (e.g., Roberts et al. 2001) have not paired water from control containers with samples stored in household environs. Another potential problem with previous studies is that most rely on self-reported data on water source, which might introduce bias because people may misrepresent where they get their water because of recall or other forms of interview bias (Wright et al. 2004).

To address some of these methodologic issues and to explore some of the heterogeneity seen in previous studies, we carried out a controlled experiment to compare microbiological contamination of drinking water between the source and POU in northern coastal Ecuador. We sampled water from the same source at the same time as members of the study households filled their containers. We also followed this water over time, comparing microbiological contamination of water in containers filled at the time of the visit that were stored in the household with containers filled with the same source water that were stored in controlled conditions. In addition to assessing differences in water quality between source and POU samples and between POU and control samples, we also explored the influence of a series of potential covariates on determining water quality in these samples.

## Materials and Methods

### Study area

**Figure 1:**
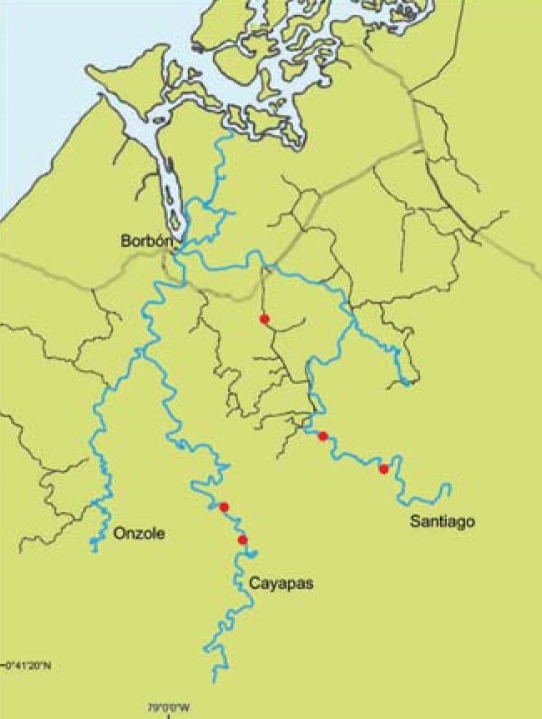
Map of study region in northwestern Ecuador. The Santiago, Cayapas, and Onzole Rivers converge in the main town of Borbón (population 5,000). Red circles represent study villages.

We carried out this study in northern coastal Ecuador, in the province of Esmeraldas, Canton Eloy Alfaro, and in five villages situated along the Santiago, Cayapas, and Onzole rivers (Figure 1). Two of these villages rely on simple piped water systems that transport untreated surface water, two rely on surface water from fast-flowing rivers, and one relies on surface water from a small stream. In addition to their primary water source (tap or surface water), some villagers also use simple wells or collect rainwater as source waters for drinking.

Little sanitation infrastructure exists in these communities. Our field staff carried out surveys about sanitation and water use practices of > 1,000 households in 21 villages in this region, including all of the villages reported in this study. According to these surveys, although some people use private or community latrines, 60% of people dispose of human waste out in the open, by digging a hole, or directly into the river. This same river serves as the primary water source for 68% of households, and 60% of households reported drinking their water without treating it. High rates of diarrheal disease have been observed in this study area (Eisenberg et al. 2006; Vieira et al. 2007).

We collected village and household water samples in conjunction with a case–control study of diarrhea incidence in each village, which determined which households to sample. Over the course of each 15-day visit, all cases of diarrhea (defined as three loose stools in a 24-hr period) were identified through daily visits to the households in the community by local field technicians and community health workers. A household was considered a case if one or more members experienced diarrhea during the 15-day period. For every household with a case of diarrhea, at least one control household without a case of diarrhea was randomly selected. Samples of both household drinking water and source waters were collected for case and control households. Sample collection and processing took place between March 2005 and March 2006. All contact with human subjects complied with applicable U.S. requirements and international regulations, as approved by the institutional review boards of the University of California, Berkeley, and the Universidad San Francisco de Quito (Quito, Ecuador). Study participants gave oral informed consent before participation in the study.

### Sample processing

Samples were collected in a manner consistent with how users collect and serve their drinking water. Container openings and taps were not sterilized before sampling. All samples were collected in Whirl-Pak bags (NASCO Corp., Fort Atkinson, WI) and kept on ice until processed. Samples were held for an average of approximately 9 hr, but were always processed within a maximum of 24 hr.

Because of the remoteness of the field sites, some modifications of standard laboratory methods were necessary. Culturing was carried out in a field laboratory set up in a house or health dispensary in the villages in which samples were collected; a modular field hood made from Plexiglas and metal was used to avoid contamination. Enterococci plates were incubated at 41 ± 2°C using an egg incubator and generator where electricity was not available, and *Escherichia coli* plates were incubated at ambient temperatures (30 ± 2°C). Agar plates were poured at a microbiology laboratory in Quito, wrapped individually in Parafilm, packaged in plastic bags, and then transferred to the field site in coolers within 5 days.

Water quality was evaluated for microbiological contamination using membrane filtration. A sample of water was passed through a 47-mm-diameter 0.45-μm cellulose filter (Millipore Inc., Billerica, MA) and then rinsed with a phosphate-buffered saline solution (pH 7.4 ± 0.2) before being transferred to a growth medium plate. The stainless-steel membrane filtration apparatus (Millipore) was dipped in alcohol, flame sterilized, and cooled between each sample. *E. coli* was detected using MI agar (BD Difco, Franklin Lakes, NJ) prepared according to U.S. Environmental Protection Agency (EPA) Method 1604 (U.S. EPA 2002b); enterococci was detected with mEI agar (BD Difco) prepared according to U.S. EPA Method 1600 (U.S. EPA 2002a). Plates were counted after 24 hr of incubation.

Logistical limitations of the research context required optimization of protocols for testing the water samples. Serial dilutions were not possible because of the multiple tubes that would have been required to transport to the field. Thus, in most cases we processed only one volume for each sample. In most samples, a volume of 10 mL was filtered through the membrane filtration unit, but to minimize nondetectable results, if a sample was suspected of being particularly clean (rainwater, treated drinking water), a volume of 50 mL was filtered. To minimize the number of results too numerous to count (TNTC), if a sample was suspected of being particularly contaminated (based on previous samples from the same source), a smaller volume (usually 5 mL, but in a few cases 1 mL) was filtered. The number of colony-forming units (CFUs) was normalized by the volume of water processed, and multiplied by 100 to get a standardized total count per 100 mL. We included nondetects in the analysis as one-half the lower detection limit; a total of 3% of enterococci tests and 6% of *E. coli* tests had a nondetectable result. We assigned a value of 450/plate to the TNTC results because the highest reported count was 400/plate; a total of 5% of enterococci tests and 7% of *E. coli* tests had a TNTC result. Note that these values for the lower and upper detection limits varied depending on the volume of water processed. Possible results therefore ranged from 1 CFU/100 mL (halfway between zero and the lower detection limit of 1 CFU/50 mL) and 9,000 (the upper limit of 450 CFU/5 mL × 100 mL).

### Analysis

**Figure 2:**
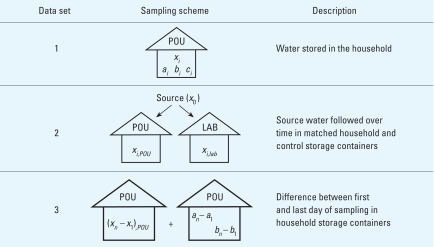
Overview of sampling schemes and data sets used in the analysis. POU includes household samples. *a*, *b*, and *c* refer to household storage containers already filled with water at the time of the initial visit to the household (some of these were followed over time); *x*, containers filled at the same time as the control container, for which a source sample was also collected (these were all followed over time); *X_i,POU_*, containers that were stored in the household; *X_i,lab_*, containers that were stored under controlled conditions. Subscripts refer to the day of sampling in the household.

Samples were collected from source waters (surface water, well, tap, rain) and POU storage containers within the households. Figure 2 shows a schematic of the sampling scheme used to assemble the three data sets for the analysis. We carried out all analyses using Stata 9.0 (StataCorp LP, College Station, TX).

#### Data set 1: water stored in the household

During the visits to the five villages, 45 of 390 total households experienced a case of diarrhea. Water was sampled from a total of 39 case households and 82 control households. In all case and control households, between one and three water containers were sampled on the first visit to the household, depending on the number of storage containers available at the time of visit. If the household had three or fewer containers with stored water, all were sampled; if it had more than three containers, three were sampled. We merged these data with all other samples taken from households to create data sets 2 and 3 for a complete data set of all samples taken from containers stored in the household.

Using this data set, we analyzed various covariates using linear regression to estimate their effects on log indicator concentrations (CFU/100 mL). The covariates we considered included community- and household-level variables: community size (number of houses per village), community sanitation (percentage of individuals in the village who stated that they used latrines or flush toilets), and crowding (number of people living in the household at the time of the visit). We also included several container-level variables: water source (rain, well, piped, river, or small stream), treatment (none, boiled, chlorinated, or left to settle), container type (small- vs. large-mouthed), covered (whether or not the container was covered or capped), and storage time (number of days since the container was filled). Container mouths were classified as < 8 cm (plastic soda bottles and jerry cans) versus > 8 cm (buckets, large water barrels, and cooking pots). Using a generalized estimating equation approach (Liang and Zeger 1986), SEs of the regression coefficients were adjusted for intragroup correlation among samples collected during the same visit to a village, which represents the highest level of possible correlation among the samples. We used village visit rather than village to account for potential differences in visits for the villages that were visited more than once.

#### Data set 2: source water followed over time in matched household and control storage containers

In 59 households, water was collected from the same source at the same time as the household members filled their water containers, and a sample of this source water was also stored in a control container similar to those most commonly used in the households (a 10-gal plastic jerry can). We selected these households based on logistical considerations, mostly related to timing constraints of daily water collection and processing. Sources were visited with household members in both the morning and the afternoon, but scheduling depended on the limited capacity for sample processing each day. The household container was marked and resampled daily for 1–5 days, until the family had finished using the water collected on the day of the visit to the source. Control containers, which were kept covered and in controlled conditions in the field laboratory, were resampled in parallel. This laboratory had environmental conditions similar to those of the households [e.g., open ceilings (space between roof and the top of the wall), no air conditioning]. Control containers were sterilized with boiling water between samples. This study design allowed for a controlled assessment of die-off and recontamination events, comparing source waters with both control and household samples.

We compared geometric mean values for samples taken directly from the source, samples taken from household containers, and samples taken from control containers. Because water quality data tend to be better fit by a log-normal distribution (Helsel and Hirsch 2002), geometric means provide a better estimate of central tendency than do arithmetic means. We also calculated the mean of paired log differences for samples from sources versus control containers (representing natural attenuation), sources versus household containers (representing net attenuation in the home), and household containers versus control containers (representing in-home recontamination). We tested the significance of these paired differences with one-sided matched paired *t*-tests. Because households differed in the number of days of sampling after initial source water collection, we calculated the paired differences for each of the different number of days that passed after initial source water collection: 1 day (59 pairs), 2 days (26 pairs), 3 days (14 pairs), 4 days (5 pairs), and 5 days (1 pair) of storage in the household and under control conditions.

In addition, we carried out linear regressions to assess the effect of number of days of water storage on the three paired differences: source–control, source–household, and household–control. Because the number of pairs changed with days of storage, we used robust SEs, and to account for repeated observations of the same container pair, regression coefficients were adjusted for intragroup correlation among samples collected from the same container using the generalized estimating equation approach described above.

#### Data set 3: household water followed over time in household storage containers

Thirty-six of the household containers sampled on the initial visit to the household (that were not included in data set 2) were resampled daily for up to 5 days. The selection of these containers was dictated both by scheduling constraints for water collection and processing and by household storage of water in containers for multiple days. We merged these data with the data from the household containers of the paired samples (data set 2), beginning with the first day of sample collection in the household, to assess recontamination in households over time. In this data set, all sampling began in the household, so reductions between the source and household due to settling or die-off may have already occurred before the first day of sampling. Graphical analysis and linear regressions were carried out to assess contamination of containers over time. Because length of water storage in the container varied (geometric mean = 46.4 hr [95% confidence interval (CI), 24.0–89.8], we controlled for time of storage in the regression analyses. We stratified these results by whether or not they experienced recontamination between the first and last sampling (difference > 0 vs. < 0), as well as other covariates.

## Results

### Analysis using data set 1

Table 1 shows the overall geometric means of indicator organism concentrations in samples stratified by source and container characteristics. In the regression analyses (Table 2), community and household factors assessed (crowding, community size, and sanitation) were less informative in explaining the variability seen in the water quality outcomes than were container characteristics (container type, treatment, covered, storage time, water source). Water treatment (as reported by members of the household) was the most important explanatory variable: boiling and chlorine were significantly associated with decreased counts of indicator organisms compared with no treatment, although the effect of settling (as a reported treatment type) was not significant. We saw this significant effect of treatment despite the small percentage of water samples that had received treatment of any kind (only 22% of samples). Additionally, container type and whether or not a container was covered at the time of sampling both showed an effect for enterococci. Water source showed an effect for *E. coli*.

### Analysis using data set 2

We found source water to be significantly more contaminated than water in the household. Source waters had a geometric mean of > 200 CFU/100 mL for both enterococci and *E. coli*, whereas samples from containers stored in the household had a geometric mean of approximately 100 CFU/100 mL for both indicator organisms. Samples from control containers had even lower geometric mean counts, on the order of 80 CFU/100 mL for all samples (Table 3).

**Figure 3:**
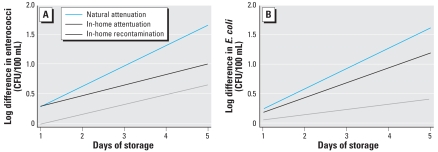
Changes over time in paired log differences for enterococci (*A*) and *E. coli* (*B*) between source, control, and household samples. Linear regressions of paired differences versus days of storage are shown for natural attenuation [source–control: (*A*), β = 0.34 ± 0.08, *p* < 0.0001; (*B*), β = 0.34 ± 0.05, *p* < 0.0001]; in-home attenuation [source–household: (*A*), β = 0.18 ± 0.13, *p* = 0.17; (*B*), β = 0.25 ± 0.10, *p* = 0.01]; and in-home recontamination [household–control: (*A*), β= 0.16 ± 0.10, *p* = 0.10; (*B*), β= 0.09 ± 0.10, *p* = 0.39]. We used a generalized estimating equation approach to adjust estimates for clustering by paired household samples, to account for autocorrelation between sampling days. Note that number of control–household container pairs decreased with number of days of storage: day 1 (*n* = 59), day 2 (*n* = 26), day 3 (*n* = 14), day 4 (*n* = 5), day 5 (*n* = 1); total *n* = 105.

The analysis of differences between the paired samples (Table 4, Figure 3) provides further insight. Difference between source and control samples can be considered to reflect natural attenuation of indicator organisms, due to settling or die-off. We observed a 0.3–2 (enterococci) and 0.2–1 (*E. coli*) log reduction, depending on the number of days of storage under controlled conditions. This value increased by 0.34 logs/day of storage, according to the linear regression analysis (β = 0.34; *p* < 0.0001 for both indicators). Differences between source and household samples reflect attenuation of indicator organisms in the home. We observed a 0.25–1 (enterococci) and 0.2–2 (*E. coli*) log reduction, depending on the number of days of storage under household conditions. The linear regression revealed that, for *E. coli*, in-home attenuation increased over time less than natural attenuation, 0.25 logs/day (*p* = 0.01). We observed no significant trend for enterococci (β = 0.18; *p* = 0.17). Last, differences between household and control containers represent total recontamination under household conditions. Household containers had on average 0.05–1 (enterococci) and 0.06–0.6 (*E. coli*) log higher indicator counts than did control containers. These differences did not increase significantly over time according to a linear model (enterococci, β = 0.16, *p* = 0.10; *E. coli*, β = 0.09, *p* = 0.39). Source waters had significantly higher concentrations of indicator organisms than did control containers at all time points and significantly higher concentrations than did household containers through day 3 only. When all sample pairs were considered, samples from household containers did not have significantly higher concentrations than did samples from control containers at any time point.

There was significant heterogeneity in the occurrence of recontamination among households; only 58% (enterococci) and 46% (*E. coli*) of household–control pairs showed more contamination in the household than in the control container after 1 day of storage. For those households that exhibited recontamination (defined as having a difference between household and control containers > 0 during a given time period), we observed an average 0.5–1.3 (enterococci) and 0.6–1.2 (*E. coli*) log increased level of contamination in household versus control containers, depending on number of days of storage; and no significant reductions were observed between the source and the household.

### Analysis using data set 3

**Figure 4:**
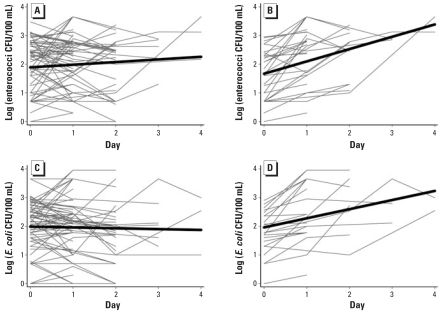
Contamination with enterococci (*A* and *B*) and *E. coli* (*C* and *D*) over time within households for all containers (*A* and *C*) and only for containers exhibiting recontamination (difference between first and last sampling > 0; *B* and *D*). Gray lines indicate individual containers, and black lines show the regression fit.

We also saw evidence of recontamination in the home in the household containers followed over time, although again, this did not occur in all cases. We observed increasing contamination between the first and last day of sampling in 46.4% (enterococci) and 32.8% (*E. coli*) of these containers. Most of the containers resampled over time were sampled only 1 day apart, either because the family had finished the water in the container or because of logistical difficulties in revisiting the house. Between the first and second day of sampling, 42.8% (enterococci) and 45.5% (*E. coli*) of containers increased in level of contamination. Figure 4 shows the overall trend for all containers, as well as only for containers that experienced recontamination (those with a difference between the first and last sampling > 0). To explain some of the heterogeneity seen in recontamination of containers, we carried out regressions of log indicator counts (CFU/100 mL) against days of storage in the household. The regression predicts a log increase in contamination over 4 days of 1.28 ± 0.49 (*E. coli*) and 2.04 ± 0.45 (enterococci) for containers experiencing recontamination. Of all the covariates assessed, the slope coefficient of the regression of large-mouthed containers most closely matches the slope coefficient of the regression for recontaminated samples only, and the odds of recontamination given a large mouth was consistently high [odds ratio (OR) = 10.7 (95% CI, 4.10–30.83) for enterococci and 3.2 (1.40–7.24) for *E. coli*] (Table 5).

## Discussion

To our knowledge, this is the first study to evaluate contamination between source and POU drinking water by sampling the same source water as collected by households in real time, and to follow its fate over the course of several days of storage within a household. It is also the first study to use paired controls to assess changes in contamination levels over time in the home. We found significantly higher concentrations of indicator organisms in source waters than in water stored in household or controlled conditions (Table 3). We observed significant natural attenuation of indicator organisms between source waters and controlled storage conditions on every day of observation, and significant in-home attenuation from the source of drinking water to its POU through the third day of storage in the home (Table 4). However, approximately half of households sampled showed an increase in concentrations of indicator organisms, representing recontamination during storage and use.

When we considered all samples, only natural attenuation significantly increased with days of storage for both indicators; in-home attenuation significantly increased with storage time only for *E. coli* (Figure 3). Averaged across all samples, in-home attenuation was significant until day 3, and we saw no significant in-home recontamination. However, in just those households that exhibited recontamination, we observed no in-home attenuation, and significant recontamination ranged from 0.5 to 1.3 (enterococci) and from 0.6 to 1.2 (*E. coli*) average log increase (Table 4). In these households, it appears that recontamination in the home made up for the reductions in indicator counts observed between the source and the household. Household members may have treated water containers differently under frequent observation, which may have led to an underestimate of the extent of recontamination in the household setting if containers were kept safe from contamination.

Given the trend reported in the literature showing a tendency for household water samples to be more contaminated than the source waters from which they were drawn (Wright et al. 2004), the recontamination we observed in the home was expected. The higher overall levels of contamination observed at the source, on the other hand, contradict this trend. This result is not unprecedented, however, and likely reflects the poor quality of source water in our study communities. In situations where source waters have low concentrations of indicator bacteria, decreased counts because of postcollection settling and/or die-off may be less likely to be observed. Studies that have compared recontamination under variable initial conditions have shown that the quality of source water affects the extent of recontamination observed in the home. For example, in a study in Venda, South Africa, Verweij et al. (1991) observed a 10- to 15-fold increase in fecal coliform counts between source and storage in water collected from boreholes, but in water samples from unprotected springs, which exhibited high initial coliform counts (~ 300 CFU/100 mL), they observed a 2-fold decline in counts over 4 hr of storage. Musa et al. (1999) found contrasting results for different types of communities in a study in northern Sudan. In rural villages and nomadic areas, where people depended on poor-quality (> 100 CFU/100 mL) source waters, fecal coliform counts were lower in storage containers than at the source, whereas in three peri-urban communities, where municipal source water was of reasonable quality (< 10 CFU/100 mL), fecal coliform counts were significantly higher in home storage containers, suggesting contamination in the household. Only the result showing recontamination in the household was reported in the review by Wright et al. (2004).

Wright et al. (2004) reported that water-quality deterioration from the source to the POU was greater for studies of uncontaminated water sources, but most of the studies in that review had high initial water quality (i.e., low counts of indicator organisms) at the source. Recontamination was also less pronounced in homes with poorer-quality source water. Within any given population, there often appears to be a subset of households in which the quality of stored water improves compared with the quality of source water (Wright J, personal communication). For example, although VanDerslice and Briscoe (1993) observed a net increase in fecal coliform counts in more than half of source–household sample pairs, they observed a net decrease in counts in 16% of households and no net change in 32%. Our methods of sampling water concurrently with household members and following these particular containers over time eliminates the possibility for bias in reporting of levels of contamination at the source, and this elimination of bias might partially explain why our results differ from many others reported in the literature.

Postcollection reductions in microbial contamination have also been observed in other studies conducted under controlled conditions. Tomkins et al. (1978) observed a marked fall in coliform counts after overnight storage in earthenware containers in a study in northern Nigeria where rural villagers relied on water from both protected and unprotected wells. Mazengia et al. (2002) reported significant reductions in bacterial loads in water urns stored in a laboratory setting compared with the source wells from which they were drawn. These reductions corresponded with declines in turbidity. In one study in rural South Africa, type of container was shown to affect rates of removal of organisms during storage: indicator organisms persisted in borehole water in polyethylene containers longer than they did in galvanized steel containers (Momba and Notshe 2003). Studies of organisms resident in water containers would further elucidate these factors.

The observed reductions in bacterial loads could be due to settling of organisms to the bottom of storage containers or die-off of these organisms caused by predation by other microorganisms, lack of nutrients, or other factors contributing to inhospitable conditions in the container. This is an important distinction, because organisms that settle out could become resuspended and consumed, thus maintaining their ability to cause infection, whereas die-off of organisms would imply loss of infectivity. In a study in Malawi, shaking of containers led to a 3-fold increase in detection of indicator organisms in unimproved buckets, suggesting that bacteria that had settled to the bottom of the storage container were still viable on resuspension (Roberts et al. 2001). Future studies should focus on distinguishing between removal and inactivation in storage containers, both in the water column and in sediment, as well as resuspension. Furthermore, the behavior of indicator organisms should be compared with that of actual pathogens. A key factor influencing removal is likely to be the particle association of indicators and pathogens, and the settling velocity of those particles. Given the high turbidity in source waters in the present study, settling within containers in the home likely explains at least part of the reduction in bacterial loads we observed. Unfortunately, we did not collect specific turbidity data for these samples, but this would be a useful avenue for future research.

After the initial reductions between source and POU, we observed an increase in contamination in about half of the households, as can be observed in Figures 3 and 4 and as evidenced by differences between household and control samples (Tables 3 and 4). Because regrowth of indicator organisms is known to occur in the tropics (Toranzos 1991), regrowth could have been misconstrued as recontamination. However, the consistent decreases of indicators we observed in the control containers suggest either that regrowth did not occur, or that it was masked by die-off and/or settling. Even if regrowth of indicator organisms had occurred, the use of control containers allowed us to assess the total change in contamination occurring within the household, by using the difference between indicator counts in household and control containers. Because most studies do not take such natural reductions into account, they might actually underestimate the extent of recontamination in the household environment, or miss it altogether. In this study, high concentrations of indicator bacteria might have masked recontamination in the household had we not used control containers.

The results of the regression analyses (Table 2) suggest that water treatment by boiling and chlorination was associated with reduced contamination. Larger mouths and uncovered containers were associated with decreased water quality as indicated by enterococci. All water sources were significantly more contaminated than rainwater as assessed with *E. coli*, but only river water was significantly more contaminated as assessed with enterococci.

Large-mouthed containers had a significantly higher odds of recontamination than small-mouthed containers, and the slope of regressions of indicator concentrations for large-mouthed containers most closely matched that of containers exhibiting recontamination overall, suggesting that mouth size may be a large factor in determining whether a container becomes recontaminated in the home (Table 5). These results are consistent with previous studies showing that factors related to the container, such as large versus small mouth and covered or uncovered, are key factors in determining quality of stored water (Mintz et al. 1995).

With the growing recognition of the issue of household recontamination, many authors have recommended focusing interventions on improving water quality at the POU rather than improving water supply or water quality (Clasen and Bastable 2003; Mintz et al. 2001; Reiff et al. 1996). A wide range of interventions aimed at improving drinking water in the home are being implemented, including improving vessels (e.g., Hammad and Dirar 1982; Mintz et al. 1995; Roberts et al. 2001), and decontaminating drinking water using chlorine (Mintz et al. 1995, 2001; Quick et al. 1999; Reiff et al. 1996), sunlight (Conroy et al. 1999), ceramic filtration (Clasen et al. 2006), sand filtration (Stauber et al. 2006), coagulation plus chlorination (Rangel et al. 2003), and ultraviolet light (Brownell et al. 2008). Taking into account initial source water quality in a given region is important for determining the most appropriate strategy for in-home decontamination. For example, in the region examined in this study, coagulation followed by chlorination might be most effective in reducing pathogen concentrations because the high-turbidity waters might reduce the efficacy of direct chlorination and solar disinfection, and rapidly clog ceramic filters. Introducing containers with smaller mouths or a spigot would also decrease the potential for recontamination in the home.

Future studies could be enhanced by the collection of behavioral and other data related to potential recontamination events, such as frequency of water access, method of water access, location and height of containers, presence of a spigot, and exposure to sunlight. It would also be extremely useful to study the effects of turbidity of source waters on household water quality and natural attenuation.

Although water treatment at the POU has been shown to be an effective strategy in intervention trials, this effect is not universally observed (Clasen et al. 2007; Fewtrell et al. 2005). Initial source water quality may explain some of the heterogeneity in the effectiveness of POU interventions. The results of the present study suggest that surface source waters are more contaminated than water in the homes in this region, and in some cases home contamination may be a smaller factor compared with initial source water quality in determining the quality of drinking water in the home. Thus, characteristics both at the community level (initial source water quality) and at the household level (factors affecting probability of recontamination) are important in determining ultimate drinking water quality.

In our study villages, we often observe children and adults drinking water directly from the stream. POU water treatment assumes traditionally that the POU is the household, when this may seldom be the case, particularly for some family members. In areas such as the one we studied, with poor sanitation and poor source water quality, where villagers drink straight from the stream, improving water quality in the home may not be sufficient to break the cycle of transmission of waterborne pathogens. Poor sanitation around a household can provide a direct route of contact with fecal contamination and can also be a source of contamination of the surface water supply. Although recent reviews have found little or no evidence that efficacy of water-quality interventions is related to levels of sanitation (Clasen et al. 2007; Fewtrell et al. 2005), others have suggested that the efficacy of household water-quality interventions depends on the level of sanitation within the target community (Esrey and Habicht 1986; Gundry et al. 2004; VanDerslice and Briscoe 1995). Furthermore, intervening only at the household level ignores the health risks of bathing in contaminated waters. Given the nonlinear nature of transmission of waterborne diseases and the complex set of interdependent pathways by which enteric pathogens are transmitted (Eisenberg et al. 2007), focusing solely on household interventions without reducing the sources of contamination in the community is not likely to be as effective as implementing integrated control strategies that include sanitation and improvement of water quality at the source through improved sanitation. In areas where initial source water quality is poor, in-home water treatment and safe water storage may need to be augmented by efforts to improve sanitation and/or source water quality.

## Figures and Tables

**Table 1:** Geometric means of indicator organism concentrations (CFU/100 mL) in household containers, stratified by various characteristics.

Characteristic	No.	Enterococci	*E. coli*
Container type			
Small-mouthed	372	74	41
Large-mouthed	260	110	65
*p*-Value		0.001	0.68
Container covered?			
Yes	415	62	45
No	228	161	69
*p*-Value		< 0.0001	0.009
Water treatment			
None	500	122	81
Boiling	48	14	11
Chlorine	42	26	19
Let settle	6	48	120
*p*-Value		0.05	0.25
Water source			
Rain	104	74	9
Well	25	64	45
Tap	259	86	51
River	122	242	272
Stream	117	58	91
*p*-Value		0.003	< 0.0001

Reported *p*-values test equality of means using *t*-tests (binary variables) or analyses of variance (variables with multiple categories).

**Table 2:** Effects of covariates on quality of water stored in household containers as measured using log values of *E. coli* and enterococci concentrations (CFU/100 mL) as outcome variables.

				Enterococci vs. variable		*E. coli* vs. variable	
Variable	Level	Description	No.	Unadjusted	Adjusted	Unadjusted	Adjusted
Crowding	Household	No. people in household	155				
β				0.06	0.00	0.03	−0.03
SE				0.01	0.01	0.02	0.01
*p*-Value				0.002	0.970	0.253	0.155
Community size	Community	No. houses in village	5				
β				0.00	0.00	0.00	0.00
SE				0.00	0.00	0.00	0.00
*p*-Value				0.58	0.58	0.97	0.28
Sanitation	Community	Sanitation index	5				
β				0.01	0.01	0.00	0.01
SE				0.01	0.01	0.00	0.00
*p*-Value				0.288	0.029	0.827	0.097
Covered	Container	1 = Covered	415				
		2 = Uncovered	228				
β				0.41	0.29	0.16	0.08
SE				0.07	0.07	0.11	0.08
*p*-Value				0.000	0.008	0.191	0.358
Water source	Container	1 = Rain	104				
		2 = Well	25				
β				−0.06	−0.37	0.71	1.19
SE				0.28	0.46	0.28	0.12
*p*-Value				0.831	0.448	0.040	0.001
		3 = Piped	259				
β				0.07	0.14	0.75	0.90
SE				0.12	0.17	0.16	0.12
*p*-Value				0.590	0.430	0.002	0.001
		4 = River	122				
β				0.51	0.39	1.48	0.95
SE				0.19	0.16	0.30	0.07
*p*-Value				0.497	0.052	0.002	< 0.0001
		5 = Small stream	117				
β				−0.10	0.09	1.01	1.18
SE				0.14	0.33	0.14	0.31
*p*-Value				0.497	0.789	< 0.0001	0.018
Treatment	Container	0 = None	500				
		2 = Boiled	48				
β				−0.94	−0.97	−0.88	−1.56
SE				0.09	0.14	0.15	0.16
*p*-Value				< 0.0001	< 0.0001	0.001	0.001
		3 = Chlorination	42				
β				−0.67	−0.92	−0.62	−1.10
SE				0.26	0.04	0.17	0.18
*p*-Value				0.028	< 0.0001	0.007	0.004
		4 = Left to settle	6				
β				−0.40	−0.19	0.18	0.33
SE				0.56	0.77	0.32	0.44
*p*-Value				0.489	0.815	0.604	0.498
Container	Container	1 = Small mouth	372				
		2 = Large mouth	260				
β				0.17	0.37	−0.21	0.23
SE				0.13	0.11	0.12	0.12
*p*-Value				0.230	0.013	0.127	0.124
Storage time	Container	No. of days since filled	602				
β				0.00	0.00	0.00	0.00
SE				0.00	0.00	0.00	0.00
*p*-Value				0.021	0.006	0.028	0.011

Unadjusted values report the results of univariate analyses; adjusted values report the results of multivariate analysis, including all covariates in the model. SEs of the regression coefficients were adjusted for intragroup correlation among samples collected during the same visit to a village using a generalized estimating equation approach.

**Table 3:** Overall levels of contamination (cfu/100 mL) at the source and in household and control containers [geometric mean (95% CI)].

	No.	Enterococci	*E. coli*
Source	59	227.9 (139.5–372.4)	227.1 (144.6–356.9)
Household	105	103.7 (70.7–152.1)	113.4 (80.8–159.0)
Control	105	80.8 (54.3–120.1)	83.8 (57.8–121.5)

**Table 4:** Mean (± SE) paired log differences between water samples from source and control containers (natural attenuation), source and household containers (in-home attenuation), and household and control containers (in-home recontamination).

	Enterococci					*E. coli*				
	Day 1	Day 2	Day 3	Day 4	Day 5	Day 1	Day 2	Day 3	Day 4	Day 5
All samples										
No.	59	26	14	5	1	59	26	14	5	1
Natural attenuation	0.30 ± 0.84	0.52 ± 1.35	1.18 ± 1.14	1.03 ± 0.78	1.92	0.24 ± 0.80	0.63 ± 0.99	1.07 ± 0.87	1.11 ± 0.41	1.12
	(*p* = 0.004)	(*p* = 0.029)	(*p* = 0.001)	(*p* = 0.021)	—	(*p* = 0.013)	(*p* = 0.002)	(*p* = 0.0003)	(*p* = 0.002)	—
In-home attenuation	0.25 ± 0.87	0.48 ± 1.26	1.05 ± 1.32	0.12 ± 1.63	0.75	0.18 ± 0.84	0.47 ± 1.09	0.80 ± 1.18	0.51 ± 1.08	1.93
	(*p* = 0.014)	(*p* = 0.031)	(*p* = 0.005)	(*p* = 0.44)	—	(*p* = 0.05)	(*p* = 0.020)	(*p* = 0.012)	(*p* = 0.18)	—
In-home recontamination	0.05 ± 0.67	0.04 ± 0.90	0.13 ± 0.70	0.91 ± 1.13	1.18	0.06 ± 0.79	0.16 ± 0.69	0.26 ± 0.92	0.60 ± 0.93	−0.70
	(*p* = 0.30)	(*p* = 0.41)	(*p* = 0.25)	(*p* = 0.07)	—	(*p* = 0.28)	(*p* = 0.12)	(*p* = 0.15)	(*p* = 0.11)	—
Recontaminated samples only										
No.	34	11	9	4	1	27	15	7	3	0
Natural attenuation	0.43 ± 0.86	0.90 ± 1.07	0.95 ± 1.33	0.89 ± 0.82	1.92	0.49 ± 0.73	0.66 ± 0.67	0.97 ± 0.93	1.08 ± 0.54	—
	(*p* = 0.003)	(*p* = 0.010)	(*p* = 0.03)	(*p* = 0.06)	—	(*p* = 0.001)	(*p* = 0.001)	(*p* = 0.02)	(*p* = 0.04)	
In-home attenuation	−0.04 ± 0.85	0.16 ± 1.13	0.46 ± 1.21	−0.44 ± 1.21	0.75	−0.18 ± 0.81	0.05 ± 0.62	0.14 ± 1.14	−0.08 ± 0.97	—
	(*p* = 0.60)	(*p* = 0.33)	(*p* = 0.15)	(*p* = 0.74)	—	(*p* = 0.87)	(*p* = 0.38)	(*p* = 0.37)	(*p* = 0.55)	
In-home recontamination	0.47 ± 0.44	0.75 ± 0.73	0.5 ± 0.34	1.32 ± 0.75	1.18	0.66 ± 0.64	0.61 ± 0.44	0.83 ± 1.0	1.16 ± 0.75	—
	(*p* < 0.0001)	(*p* = 0.004)	(*p* = 0.001)	(*p* = 0.02)	—	(*p* < 0.0001)	(*p* < 0.0001)	(*p* = 0.04)	(*p* = 0.06)	

Results are shown for all container pairs and also for just those container pairs exhibiting recontamination (in-home recontamination > 0 during that time period), stratified by the number of days of water storage in the household. *p*-Values are for one-sided matched paired *t*-tests comparing log values for sample pairs.

**Table 5:** Factors affecting recontamination.

	All containers	Recontaminated	Treated	Large mouth	Uncovered
Enterococci					
*n*	158	74	37	42	119
Slope	0.11	0.51	0.05	0.40	0.13
	(−0.06 to 0.27)	(0.28 to 0.74)	(−0.40 to 0.51)	(0.16 to 0.63)	(−0.15 to 0.21)
OR	—	—	1.27	10.7*	0.86
	—	—	(0.57 to 2.83)	(4.10 to 30.83)	(0.39 to 1.91)
*E. coli*					
*n*	158	47	37	42	119
Slope	−0.01	0.32	−0.09	0.16	0.03
	(−0.16 to 0.17)	(0.07 to 0.57)	(−0.55 to 0.38)	(−0.09 to 0.41)	(−0.15 to 0.21)
OR	—	—	0.22*	3.2*	1.26
	—	—	(0.05 to 0.68)	(1.40 to 7.24)	(0.53 to 3.22)

Values shown are slope coefficients (95% CIs) for regressions of water quality (log indicator concentrations, CFU/100 mL), controlling for storage time (hours). ORs (95% CIs) are given for odds of recontamination for treated, large-mouth, and uncovered water.

* OR with CI that does not cross 1.0.
